# Survival probability and factors associated with time to loss to follow-up and mortality among patients on antiretroviral treatment in central Kenya

**DOI:** 10.1186/s12879-022-07505-0

**Published:** 2022-06-06

**Authors:** P. Wekesa, A. McLigeyo, K. Owuor, J. Mwangi, E. Ngugi

**Affiliations:** 1 Centre for Health Solutions – Kenya, Nairobi, Kenya; 2 Division of Global HIV & TB, Centers for Disease Control and Prevention (CDC), Nairobi, Kenya

**Keywords:** Antiretroviral therapy, HIV, Kenya, Survival, Loss to follow-up, Mortality

## Abstract

**Background:**

Retention of patients who are receiving antiretroviral therapy (ART) remains a challenge especially in the setting of rapid expansion of HIV services. Retention in care remains vital to the HIV care continuum, and has been associated with viral suppression and improved survival. This study aimed to ascertain survival rates, time to loss to follow-up (LTFU) or mortality events and factors associated with time to LTFU or mortality among patients enrolled on antiretroviral therapy at health facilities in central Kenya.

**Methods:**

This was a retrospective cohort study among patients initiated on ART between 2004 and 2012 in central Kenya. Demographic characteristics, clinical characteristics and outcomes data were analyzed using Stata version 15.1. Competing risks regression analysis and cummulative incidence functions were used to estimate survival.

**Results:**

A total of 31,346 patients were included, of whom 65.6% were female, 76.0% were aged between 20 and 50 years old, and 38.9% were diagnosed at WHO stage III. At 36 months, overall retention was 68.8%, LTFU was 27.1%, and mortality was 4.1%. The total person-years of follow up was 74,986. The incidence rate of LTFU was 9.99 per 100 person years for a total of 9383.25 person-years of follow up. The mortality rate was 1.25 per 100 person years for a total of 875.5 person-years among those who died. The median time to LTFU was 11 months (IQR 3–22) while median time to death was 3 months (IQR 0–13). Men, unmarried patients, patients presenting with advanced HIV, not on TB treatment, and enrolled into the HIV program in later cohorts, had a shorter time to mortality and LTFU.

**Conclusion:**

Our study demonstrated evidence of scale-up of HIV treatment programs in central Kenya. While most patients were enrolled at an advanced WHO clinical stage, overall 36-month mortality remained low, but occurred earlier during follow-up. Cohort LTFU at 36-months reduced in later years with the losses occurring within the 1st year of follow-up. Predictors of early mortality and LTFU included being male, single, separated or divorced, advanced WHO clinical stage, and among patients not on TB treatment.

## Background

Access to antiretroviral therapy (ART) among people living with HIV (PLHIV) in sub Saharan Africa (SSA) has increased over the years [1]. Globally, there were approximately 37.7 million PLHIV, with 27.5 million receiving ART in 2020 [2]. In Kenya, there were 1.4 million PLHIV with an estimated 86% receiving antiretroviral therapy in 2020, following significant progress reflected in a 53% reduction in new infections and a 63% reduction in AIDS-related mortality from 2010 [2, 3]. Majority of patients receiving HIV treatment services in Kenya are reported to be women, receiving treatment regimens that have evolved from the initial nucleoside reverse transcriptase inhibitor based backbone to the current optimized integrase inhibitor-based regimens per national guidelines [4–7]. However, retention of patients on lifelong ART remains an implementation challenge [8]. Retention at 36 months of follow-up have been reported as 64.4% in Zimbabwe, at 55.4% and 68.6% in Kenya and 65.0% in Africa [9–12].

Retention in care (RIC) remains vital to the HIV care continuum, and has been associated with viral suppression and improved survival. Low retention rates during treatment is predominantly a result of loss to follow-up (LTFU) in HIV programs within sub-Saharan Africa (SSA) [13, 14]. Findings from a systematic review reported LTFU rates of 22.6%, 25.0% and 29.5% at 12, 24 and 36 months respectively for cohorts from countries within SSA [10]. A study in South Africa reported an incidence rate of LTFU of 10.3 per 100 person-years in the 1st year on ART and increased to 40.5 per 100 person-years in the 8th year of ART [15]. This contrasts with LTFU incidence rates reported in developed countries at 4.2 per 100 person-years reported among ART experienced participants by the AIDS Clinical Trials Group (ACTG) Longitudinal Linked Randomized Trials (ALLRT) prospective cohort of 4630 participants [16] and, 3.5 per 100 person years among 1007 patients in France between 1997 to 2006 [17].

Conversely, the incidence rate for mortality reported in developing countries has been lower compared to that of LTFU. A study conducted in Asia reported a mortality incidence rate of 1.34 per 100 person-years among 8305 patients with over 26,217 person-years of follow-up [18] while a study conducted in Ethiopia reported a mortality incidence of 1.75 per 100 person-years [19].

Few studies in Kenya have focused on the survival analysis of patients on follow-up after ART initiation. Further, little data exists regarding the factors associated with time to LTFU and time to mortality. We conducted this analysis to investigate survival probability, time to LTFU and mortality as well as factors associated with time to survival among ART patients on follow-up in Kenya.

## Methods

### Study setting and period

Centre for Health Solutions—Kenya (CHS), supported HIV Care and Treatment services in five counties in central Kenya. HIV care and treatment services were decentralized to sub county hospitals and health centers to increase the number of people living with HIV (PLHIV) accessing health services. Services are provided in comprehensive care clinics through a multi-disciplinary team composed of clinical officers, nurses, data clerks, peer educators, nutritionists, social workers, pharmacists, pharmaceutical technologists, and laboratory technologists. All health facilities offered a standard package of care that included provider initiated testing and counselling (PITC), same day enrolment into the HIV clinic, patient education and counselling, baseline CD4 count testing, ART initiation for eligible patients, treatment monitoring, structured follow-up appointments per national guidelines, comorbidity management, as well as adherence and retention support. All HIV services were offered in an integrated model that combined all services required by the patient in a one-stop-shop approach.

### Study design, population and sample

This was a retrospective cohort analysis of all patients initiating ART between 2004 and 2012 at 104 CHS supported ART and PMTCT clinics in the five counties (101 government owned and 3 private owned). The number of health facilities with data included in the study per county were 36 in Nyeri, 33 in Muranga, 26 in Kiambu, 7 in Nyandarua, and 2 in Laikipia. A total of 31,346 clients were included in the sample.

### Data sources

The data were extracted from Comprehensive Patient Application Database (CPAD), an electronic medical record system (EMR). The Ministry of Health’s (MOH) paper-based Comprehensive Care Clinic Patient Card (MOH 257) was used to record patient data during clinic visits. Data clerks based at health facilities entered this longitudinal patient-level data into the EMR, which was updated at every clinic visit. Data quality assurance activities were conducted by CHS monitoring and evaluation officers at least every 3 months to ensure data quality was acceptable.

### Outcomes and variables

Treatment outcomes were assessed at 36 months of follow-up. The outcomes of interest were retention, LTFU, and mortality. Retention was defined as alive and actively engaged in HIV care during the follow-up period. Loss to follow-up was defined as having missed a clinic attendance for 90 days or more consecutively per PEPFAR and Kenya national ART outcome monitoring guidelines. Mortality status was determined from existing documentation in the patient’s records. Patients who were still in the program were censored at 36 months. For the patients who either died or were lost to follow up, the actual time in months from initiation of treatment to the time of event of interest was recorded. Survival time was calculated for both time to death and time to LTFU. Data were abstracted from an electronic database with treatment cohorts being the comparison groups of interest. Baseline variables considered for inclusion in the models were age, sex, marital status, regimen started, WHO stage at start of ART, and TB status at enrollment visit.

### Ethics

Ethical approval was obtained before the start of the study. Local ethical approval was obtained from the Kenyatta National Hospital and University of Nairobi institutional review board (P339/06/2013). The protocol was also reviewed by the Associate Director of Science, Center for Global Health at the US Centers for Disease Control and Prevention (CDC) in line with human research protection procedures and was determined to be research, but CDC investigators did not interact with human subjects or have access to identifiable data for research purposes. Anonymity and confidentiality of data was strictly maintained. This was a retrospective study, therefore consent from study participants was waived by the Kenyatta National Hospital and University of Nairobi institutional review board. All the study procedures were done in conformity with Kenya Government, Ministry of Health, CDC, and local IRB guidelines and regulations.

### Data analysis

Patient data were descriptively presented using counts (percentages) and medians (interquartile range) as appropriate. Given the importance of each main outcome (i.e. LTFU and Mortality) for this study we conducted separate competing risks regression analyses based on Fine and Gray’s proportional sub-distribution hazards model [50]. To assess for factors associated with time to LTFU, the mortality outcome was regarded as a competing event since patients who died were no longer at risk of LTFU. A separate model was fit to assess for factors associated with time to mortality, where LTFU outcome was regarded as a competing event since they were assumed to be no longer at risk of mortality in this patient cohort. For competing-risks analysis, a Kaplan–Meier curve was inadequate because it fails to acknowledge that the competing event (death or LTFU) may never occur. In reality, the probability of death or LTFU after time zero is not equal to 1. Kaplan–Meier calculation does not take into account dependence between competing events of death and LTFU which is rectified by competing risks regression analysis [20, 21]. All variables collected (age, sex, marital status, regimen, WHO stage and TB status) were included a priori in the multivariable model. We presented cumulative incidence function (CIF) plots and reported sub-distribution hazard ratios (SHR) and respective 95% confidence intervals (CI) for each main outcome. The incidence rates of death and LTFU were reported per 100 person-years and respective 95% CI. Multivariable models used complete case analysis given the number of observations and proportions of missing data which were approximately 10% or less. Analyses were done using Stata version 15.1 (StataCorp. 2017. Stata Statistical Software: Release 15. College Station, TX: StataCorp LLC).

## Results

### Patient characteristics

A total of 31,346 patients were included in the study with their characteristics shown in Table 1. More than half were women (65.6%) and 45.9% were married. The majority of patients were aged between 36–50 years (39.6%) and 20–35 years (37.1%). Half of the patients were on a stavudine-based (D4T) ART regimen (50.8%) and 3.9% were on tuberculosis (TB) treatment for TB/HIV coinfection at enrollment. Majority were classified as WHO Stage III/IV (45.7%) and Stage II (35.9%).

**Table 1:** Characteristics of ART patients in central Kenya between 2004 and 2012

Participant characteristics	Total	Retained	LTFU	Dead	p value
n (%)	31,346 (100.0)	21,576 (68.8)	8495 (27.1)	1275 (4.1)	
	n (%)	n (%)	n (%)	n (%)	
Age at enrollment (N = 31,346)					< 0.001
0–4	1533 (4.9)	1078 (5.0)	417 (4.9)	38 (3.0)	
5–9	1466 (4.7)	1100 (5.1)	323 (3.8)	43 (3.4)	
10–14	842 (2.7)	621 (2.9)	189 (2.2)	32 (2.5)	
15–19	500 (1.6)	342 (1.6)	147 (1.7)	11 (0.9)	
20–35	11,630 (37.1)	7534 (34.9)	3659 (43.1)	437 (34.3)	
36–50	12,422 (39.6)	8822 (40.9)	3061 (36.0)	539 (42.3)	
51 and above	2953 (9.4)	2079 (9.6)	699 (8.2)	175 (13.7)	
Sex, (n = 31,346)					< 0.001
Male	10,791 (34.4)	7208 (33.4)	3002 (35.3)	581 (45.6)	
Female	20,555 (65.6)	14,368 (66.6)	5493 (64.7)	694 (54.4)	
Marital status (n = 27,497)					< 0.001
Single	6964 (25.3)	4641 (24.4)	1973 (27.0)	350 (30.9)	
Married	12,622 (45.9)	9045 (47.5)	3144 (43.0)	433 (38.3)	
Divorced	4554 (16.6)	2949 (15.5)	1402 (19.2)	203 (17.9)	
Widowed	3357 (12.2)	2421 (12.7)	790 (10.8)	146 (12.9)	
Regimen (n = 29,192)					< 0.001
D4T	14,823 (50.8)	9861 (48.6)	4200 (54.3)	762 (64.1)	
AZT	6363 (21.8)	4642 (22.9)	1508 (19.5)	213 (17.9)	
TDF	6578 (22.5)	4764 (23.5)	1631 (21.1)	183 (15.4)	
ABC	1428 (4.9)	1007 (5.0)	391 (5.1)	30 (2.5)	
WHO stage at enrollment (n = 29,623)					< 0.001
Stage 1	5471 (18.5)	4082 (19.9)	1303 (16.4)	86 (7.1)	
Stage 2	10,622 (35.9)	7646 (37.3)	2663 (33.6)	313 (25.7)	
Stage 3	11,523 (38.9)	7637 (37.3)	3261 (41.2)	625 (51.3)	
Stage 4	2007 (6.8)	1118 (5.5)	695 (8.8)	194 (15.9)	
TB status (n = 29,262)					< 0.001
No TB signs	27,828 (95.1)	20,087 (98.7)	6898 (88.8)	843 (73.8)	
Presumptive TB	289 (1.0)	90 (0.4)	124 (1.6)	75 (6.6)	
On TB TX	1145 (3.9)	176 (0.9)	745 (9.6)	224 (19.6)	
ART cohort (n = 31,346)					< 0.001
2004	430 (1.4)	285 (1.3)	137 (1.6)	8 (0.6)	
2005	1294 (4.1)	933 (4.3)	332 (3.9)	29 (2.3)	
2006	2800 (8.9)	2073 (9.6)	633 (7.5)	94 (7.4)	
2007	4205 (13.4)	2731 (12.7)	1270 (14.9)	204 (16.0)	
2008	4553 (14.5)	3098 (14.4)	1198 (14.1)	257 (20.2)	
2009	4667 (14.9)	3297 (15.3)	1129 (13.3)	241 (18.9)	
2010	4798 (15.3)	3348 (15.5)	1230 (14.5)	220 (17.3)	
2011	4900 (15.6)	3463 (16.1)	1298 (15.3)	139 (10.9)	
2012	3699 (11.8)	2348 (10.9)	1268 (14.9)	83 (6.5)	

### Outcomes

Overall retention was 68.8%, LTFU was 27.1%, and mortality was 4.1% at 36-months as shown in Table 1. There were significant differences in the outcome by age, sex, marital status, regimen, WHO stage, TB status, and ART cohort (all p values < 0.001). At 36 months, the proportion of patients retained on ART ranged from 74.0% in 2006 to 63.5% in 2012. Loss-to-follow up was highest in 2012 (34.3%) and lowest in 2009 (24.2%). Mortality at 36 months of follow-up was highest in the 2008 ART cohort (5.6%) and lowest in the 2004 ART cohort (1.9%) as shown in Table 2.

**Table 2:** Treatment outcomes for ART patients in central Kenya between 2004 and 2012 after 36 months of follow up

Outcome	ART cohort								
	2004 n%	2005 n%	2006 n%	2007 n%	2008 n%	2009 n%	2010 n%	2011 n%	2012 n%
Retained	285	933	2073	2731	3098	3297	3348	3463	2348
	66.3	72.1	74.0	64.9	68.0	70.6	69.8	70.7	63.5
LTFU	137	332	633	1270	1198	1129	1230	1298	1268
	31.9	25.7	22.6	30.2	26.3	24.2	25.6	26.5	34.3
Dead	8	29	94	204	257	241	220	139	83
	1.9	2.2	3.4	4.9	5.6	5.2	4.6	2.8	2.2

The total person years of follow up was 74,986. The incidence rate (per 100 person-years) of LTFU was 9.99 (95% CI 9.77–10.22) for a total of 9383.25 person-years of follow up. The mortality rate (per 100 person-years) was 1.25 (95% CI 1.17–1.33) for a total of 875.5 person-years among those who died. The median time to LTFU was 11 months (IQR 3–22) while median time to death was 3 months (IQR 0–13).

### Factors associated with time to mortality over 36 months of follow-up

**Fig. 1:**
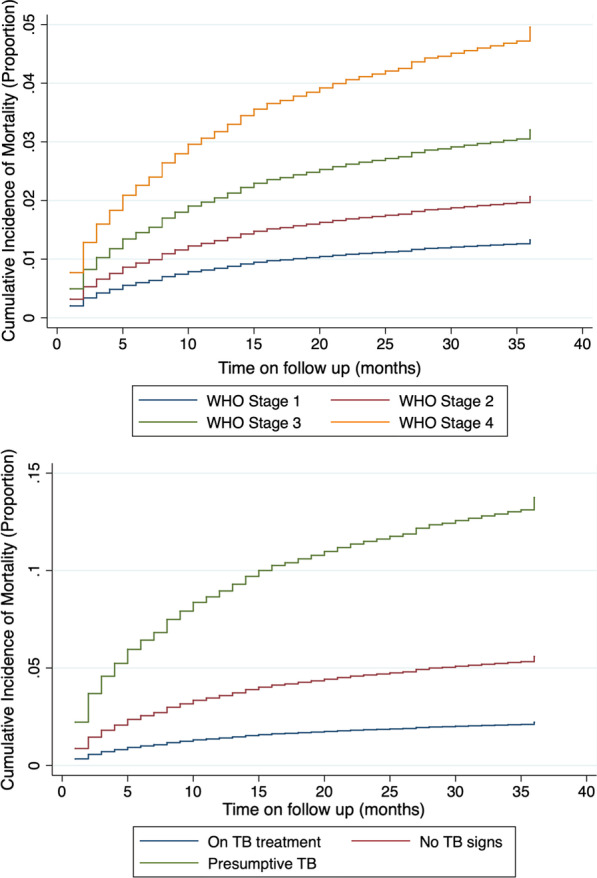
Cummulative incidence of mortality based on WHO stage and TB status

Factors associated with time to mortality on multivariable analysis included age, sex, marital status, WHO stage, TB status, and the year of cohort enrolment. There was a two times higher risk of mortality among those aged 51 years or more at enrolment (aSHR = 2.40 [95% CI 1.32–4.34], p = 0.004). Men were more likely to have shorter time to mortality compared to females, (aSHR = 1.63 [95% CI 1.38–1.91], p < 0.001), while those who were single, divorced or widowed in that order were more likely to have shorter time to mortality compared to those who were married as shown in Table 3. Mortality risk increased as the WHO stage at enrolment increased being twofold for those enrolled in WHO stage III (aSHR = 2.01 [95% CI 1.50–2.68], p < 0.001) and fourfold for those enrolled in WHO stage IV (aSHR = 4.21 [95% CI 2.98–5.96], p < 0.001). Among patients with presumptive TB, there was a sixfold higher risk of shorter time to mortality (aSHR = 6.05 [95% CI 4.91–7.45], p < 0.001) and eightfold higher odds among those with no signs of TB at enrolment (aSHR = 8.67 [95% CI 6.22–12.1], p < 0.001) compared to those on TB treatment as shown in Fig. 1. Patients initiating ART from 2007 to 2011 had higher risk of mortality using the 2004 cohort as reference, with sub-distribution hazard ratio in 2007 at (aSHR = 3.20 [95% CI 1.18–8.68], p = 0.023) peaking in 2010 at (aSHR = 4.13 [95% CI 1.50–11.33], p = 0.006) before declining in 2011 (aSHR = 3.00 [95% CI 1.07–8.46], p = 0.037). The univariable sub-distribution hazard ratio trends were similar in direction.

**Table 3:** Factors associated with time to mortality for patients on ART in central Kenya between 2004 and 2012 after 36 months of follow up

	Univariable		Multivariable	
	SHR (95% CI)	p value	SHR (95% CI)	p value
Age groups				
0–4	Ref		Ref	
5–9	1.17 (0.72–1.91)	0.529	0.84 (0.41–1.72)	0.633
10–14	1.72 (1.03–2.86)	0.037	1.01 (0.46–2.20)	0.979
15–19	0.80 (0.37–1.75)	0.575	0.54 (0.19–1.52)	0.239
20–35	1.36 (0.94–1.98)	0.108	1.42 (0.81–2.50)	0.224
36–50	1.65 (1.14–2.39)	0.008	1.59 (0.90–2.80)	0.107
51 and above	2.36 (1.59–3.51)	< 0.001	2.40 (1.32–4.34)	0.004
Sex				
Female	Ref		Ref	
Male	1.63 (1.44–1.86)	< 0.001	1.63 (1.38–1.91)	< 0.001
Marital status				
Single	1.47 (1.25–1.74)	< 0.001	1.75 (1.42–2.15)	< 0.001
Married	Ref		Ref	
Divorced	1.37 (1.13–1.67)	0.001	1.33 (1.07–1.65)	0.010
Widowed	1.31 (1.05–1.63)	0.016	1.29 (1.01–1.66)	0.044
Regimen started				
TDF based	Ref		Ref	
D4T based	1.82 (1.51–2.20)	< 0.001	1.31 (1.00–1.72)	0.050
AZT based	1.30 (1.04–1.63)	0.021	1.15 (0.87–1.51)	0.339
ABC based	0.81 (0.52–1.25)	0.335	1.21 (0.72–2.05)	0.480
Baseline WHO stage				
Stage 1	Ref		Ref	
Stage 2	1.82 (1.40–2.37)	< 0.001	1.42 (1.06–1.91)	0.020
Stage 3	3.14 (2.45–4.03)	< 0.001	2.01 (1.50–2.68)	< 0.001
Stage 4	5.42 (4.05–7.24)	< 0.001	4.21 (2.98–5.96)	< 0.001
TB status				
ON TB TX	Ref		Ref	
No signs	10.50 (7.87–14.02)	< 0.001	8.67 (6.22–12.10)	< 0.001
Presumptive TB	8.10 (6.80–9.65)	< 0.001	6.05 (4.91–7.45)	< 0.001
ART cohort				
2004	Ref			
2005	0.79 (0.34–1.79)	0.567	0.86 (0.26–2.77)	0.795
2006	1.31 (0.63–2.73)	0.464	2.29 (0.83–6.34)	0.112
2007	1.94 (0.95–3.94)	0.068	3.20 (1.18–8.68)	0.023
2008	2.13 (1.05–4.32)	0.036	3.97 (1.46–10.77)	0.007
2009	1.90 (0.93–3.85)	0.077	4.07 (1.50–11.07)	0.006
2010	1.78 (0.88–3.61)	0.112	4.13 (1.50–11.33)	0.006
2011	1.09 (0.53–2.24)	0.812	3.00 (1.07–8.46)	0.037
2012	0.89 (0.43–1.85)	0.748	2.57 (0.90–7.32)	0.078

### Factors associated with time to LTFU

**Fig. 2:**
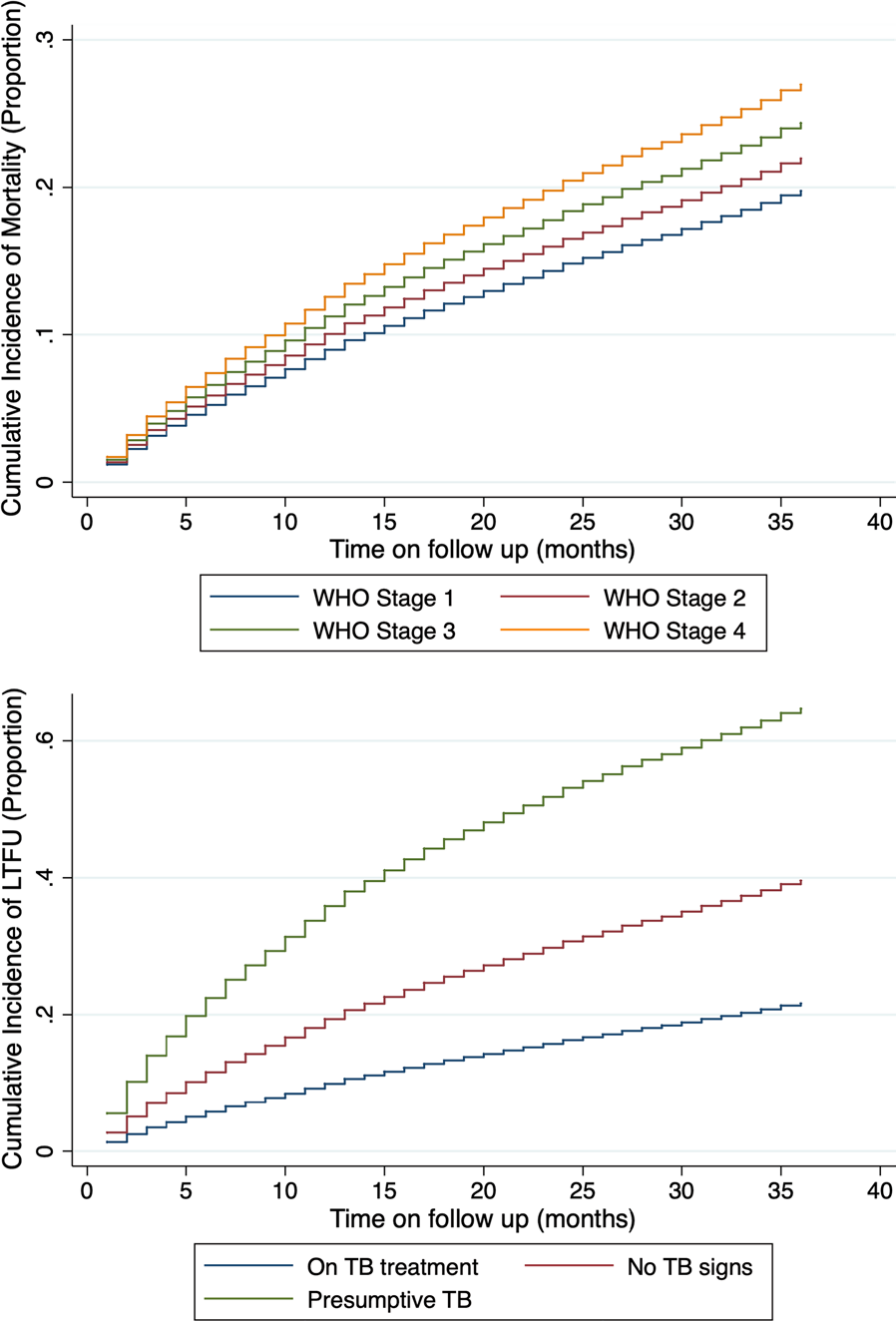
Cummulative incidence of LTFU by WHO stage and TB status

Factors associated with time to LTFU on multivariable analysis were sex, age, marital status, initial ART regimen, WHO stage, TB status, and year of cohort enrolment. Men were more likely to have a statistically significant shorter time to LTFU compared to females, (aSHR = 1.07 [95% CI 1.01–1.14], p = 0.026) as well as those aged between 20 and 35 years (aSHR = 1.26 [95% CI 1.03–1.55], p = 0.024). The single and divorced had a significantly shorter time to LTFU compared to those who were married (aSHR = 1.18 [95% CI 1.09–1.27], p < 0.001) and (aSHR = 1.26 [95% CI 1.17–1.36], p < 0.001) respectively. Using WHO Stage I as the reference group, patients with WHO Stage II (aSHR = 1.12 [95% CI 1.03–1.21], p = 0.006), WHO stage III (aSHR = 1.21 [95% CI 1.12–1.32], p < 0.001), and WHO stage IV (aSHR = 1.51 [95% CI 1.29–1.86], p < 0.001) had a significantly shorter time to LTFU. Patients on stavudine based (aSHR = 1.87 [95% CI 1.70–2.06], p < 0.001), zidovudine based (aSHR = 1.29 [95% CI 1.19–1.41], p < 0.001, and abacavir based (aSHR = 1.55 [95% CI 1.29–1.86], p < 0.001) regimens had a significantly shorter time to LTFU using TDF as reference. Patients who were presumed to have TB, (aSHR = 4.08 [95% CI 3.64–4.57], p < 0.001), or no signs of TB, (aSHR = 1.93 [95% CI 1.50–2.49], p < 0.001), also had a statistically significant shorter time to LTFU compared to those who were on TB treatment. The odds of shorter time to LTFU increased with the year of enrolment from 2007 (aSHR = 1.47 [95% CI 1.08–2.00], p = 0.015) peaking in 2012 (aSHR = 3.47 [95% CI 2.52–4.76], p < 0.001) using 2004 as reference as shown in Table 4. Figure 2 indicates the cumulative incidence of LTFU by WHO stage and TB status.

**Table 4:** Factors associated with time to LTFU for patients on ART in central Kenya between 2004 and 2012 after 36 months of follow up

	Univariable		Multivariable	
	SHR (95% CI)	p value	SHR (95% CI)	p value
Age groups				
0–4	Ref		Ref	
5–9	0.82 (0.70–0.95)	0.009	0.76 (0.59–0.98)	0.035
10–14	0.86 (0.72–1.03)	0.096	0.78 (0.59–1.04)	0.088
15–19	1.18 (0.97–1.43)	0.095	1.08 (0.81–1.44)	0.604
20–35	1.22 (1.10–1.36)	< 0.001	1.26 (1.03–1.55)	0.024
36–50	0.90 (0.81–1.01)	0.063	0.91 (0.74–1.12)	0.363
51 and above	0.86 (0.76–0.98)	0.022	0.85 (0.68–1.06)	0.139
Sex				
Female	Ref		Ref	
Male	1.03 (0.98–1.08)	0.308	1.07 (1.01–1.14)	0.026
Marital status				
Single	1.19 (1.12–1.26)	< 0.001	1.18 (1.09–1.27)	< 0.001
Married	Ref		Ref	
Divorced	1.30 (1.21–1.38)	< 0.001	1.26 (1.17–1.36)	< 0.001
Widowed	0.93 (0.86–1.02)	0.106	1.04 (0.94–1.14)	0.460
Regimen started				
TDF based	Ref		Ref	
D4T based	0.61 (0.45–0.82)	0.004	1.87 (1.70–2.06)	< 0.001
AZT based	0.48 (0.35–0.65)	0.632	1.29 (1.19–1.41)	< 0.001
ABC based	0.78 (0.45–1.36)	0.042	1.55 (1.29–1.86)	< 0.001
Baseline WHO stage				
Stage 1	Ref		Ref	
Stage 2	1.05 (0.98–1.12)	0.188	1.12 (1.03–1.21)	0.006
Stage 3	1.20 (1.12–1.28)	< 0.001	1.21 (1.12–1.32)	< 0.001
Stage 4	1.50 (1.36–1.66)	< 0.001	1.51 (1.32–1.71)	< 0.001
TB status				
ON TB TX	Ref		Ref	
No signs	1.88 (1.50–2.36)	< 0.001	1.93 (1.50–2.49)	< 0.001
Presumptive TB	4.59 (4.17–5.05)	< 0.001	4.08 (3.64–4.57)	< 0.001
ART cohort				
2004	Ref			
2005	0.73 (0.58–0.93)	0.010	0.78 (0.55–1.12)	0.175
2006	0.71 (0.57–0.88)	0.002	0.99 (0.72–1.36)	0.929
2007	1.02 (0.83–1.26)	0.837	1.47 (1.08–2.00)	0.015
2008	0.89 (0.72–1.10)	0.275	1.46 (1.07–2.00)	0.016
2009	0.81 (0.66–1.00)	0.049	1.53 (1.12–2.08)	0.007
2010	0.87 (0.71–1.07)	0.189	1.94 (1.42–2.65)	< 0.001
2011	0.90 (0.73–1.11)	0.324	2.45 (1.79–3.37)	< 0.001
2012	1.21 (0.99–1.49)	0.068	3.47 (2.52–4.76)	< 0.001

## Discussion

The majority of patients enrolled into the ART program between 2004 and 2012 were women. This reflects the national sex distribution of HIV and the awareness of HIV status among women in Kenya [4, 22]. Majority of adult patients in this study were also married, which is similar to patient populations within other sub-Saharan African countries [23, 24]. The WHO stage at diagnosis in most patients was WHO clinical stage III during the period under study. Though proportionaly lower in our study, this is similar to findings from a study in Ethiopia that reported upto 68.8% enrolment at an advanced WHO clinical stage [25]. In contrast, other studies conducted during a similar period reported diagnosis of HIV at early WHO clinical stages and at higher CD4 counts [26, 27]. The absence of TB signs was high in this study. This reflects findings from the 2016 TB prevalence survey that suggested missed opportunities for TB diagnosis at health facilities in Kenya [28]. Overall, there was growth in enrolment numbers in the period demonstrating scale-up of ART in the region.

With regards to outcomes, our study reported a 36-month retention rate of 68.8%. This was higher than that reported in a study in Zimbabwe with a 36-month retention rate of 64.4% [9]. These findings highlight the difficulties that HIV programs face in sustaining longitudinal follow-up for chronic patients. We however note that HIV treatment programs are currently implementing person-cetered interventions such as community and facility-based differentiated service delivery (DSD) models that are reported to improve retention in care [29, 30]. The isolated decline in retention for the 2012 cohort in our study could have been due to unplanned treatment interruption from election-related political instability following the 2012 general elections in Kenya [31].

Loss to follow-up decreased from 31.0% in 2004, to 22.0% in 2006 and then increased to 34.0% in 2012. This is similar to loss to follow up rates reported in literature. For example, among 88,000 adults on ART in a Tanzania HIV program, 36.0% were lost to follow-up at 36 months [32]. In a study of HIV programs in Africa and Asia, the 36 month LTFU progressively increased from 11.0 to 21.0% over 5 cohort years [33]. Rapid program expansion has been found to be a contributing factor for high LTFU, alongside decentralization of ART services, self-transfer of patients, and improved documentation of outcomes [33].

The total person years of follow up was 74,986 in this study. The incidence rate of LTFU was 9.99 per 100 person years. This incidence rate was lower than the 12.1–15.3 per 100 person years reported in Latin America and the Caribbean countries [34] but is similar to the 10.9 per 100 person years reported in South Africa [35], 10 per 100 person years in Zambia [36], and 8.2 per 100 person years in Ethiopia [37]. It was however higher than the 4.5 and 3.5 per 100 person years reported in two studies conducted in France between 1985 to 1998, and 1997 and 2006 respectively [9, 38]. The variation may be explained by differences in study setting and period, health seeking behavior, as well as documentation of transfers and deaths.

The median time to LTFU was 11 months. Factors associated with a shorter time to LTFU included being male, single, separated and widowed, age 20–35, advanced HIV at enrolment, initiation of non-TDF based ART regimens, presence of presumptive or unknown TB status, and enrolment in later cohorts. Studies in sub-Saharan Africa have reported high rates of LTFU within 6 months following ART initiation [39]. More than half the patients receiving ART in two care and treatment centers in Tanzania were LTFU within 3 months of ART initiation [40]. A study conducted in Ethiopia reported a median LTFU time of 15 months for men, and 23 months for women with nearly half being LTFU within the first 6 months and 73.0% before 1 year [41]. WHO clinical stage IV was an independent predictor of LTFU [42]. Advanced immunodeficiency at baseline was similarly reported as associated with early LTFU by the Antiretroviral Therapy in Lower-Income Countries (ART-LINC) collaboration study [43]. In contrast, a study conducted in France reported that immunosuppression at baseline was associated with a decreased risk of LTFU [9].

The 36-month mortality rate was 4.1%. The rate was lower for earlier and later cohorts years. This rate was lower than the 14.0% mortality rate reported in Tanzania at 36 months and 9.0% reported in a multi country study conducted in Africa and Asia [32, 33]. This represents evidence of low mortality rates for patients on ART during the scale up of treatment programs [44, 45]. While this presents mortality data for older treatment cohorts, we note the recent advancements in treatment, including the adoption of the ‘test and treat’ policy and more efficatious antiretroviral treatment regimens including Dolutegravir in similar settings [46, 47].

The mortality incidence in our study was 1.2 per 100 person years of follow-up with a median time to death of 3 months. This is similar to results reported in Asia at 1.34 per 100 person years among 8305 patients followed up for 26,217 person years [18], 3.46 deaths per 100 person years after 5 years of follow-up for 2388 patients in Cameroon [48] and 1.0–5.0 per 100 person years in patients followed up from 2002 to 2013 in Botswana [49]. It is however lower than the 21.2 per 100 person years reported in a different study in Cameroon [50]. The low mortality rate could have been affected by potential misclassification bias of mortality cases as LTFU. Studies from developing countries have similarly reported early mortality among patients initiating ART [51, 52]. The early mortality in this study could be due to presentation at advanced WHO clinical stages resulting in increased risk of opportunistic infections.

Patients who were men, single, divorced or widowed, aged above 51 years, enrolled at advanced WHO stage, with presumptive TB were more likely to have early mortality. Predictors of early mortality at ART initiation in Cameroon included advanced HIV and low CD4 cell count [53]. In a systematic review and meta-analysis conducted in low and middle income countries, independent factors associated with early mortality in 30 studies included low baseline CD4 cell count, male sex, advanced WHO clinical stage, low body mass index, anemia, and age greater than 40 years [33]. Yet another study reported TB coinfection, lower baseline weight, and poor treatment adherence as predictors of mortality in HIV patients [54]. This underscores the need to diagnose and initiate ART before severe immunosuppression, provide psychosocial support systems to the unmarried, screening of comorbities especially noncommunicable diseases for older patients and to establish TB status at enrollment among those with presumptive TB to ensure initiation of treatment as appropriate.

The main strength of this study is the large amount of data available over a 9 year period used for the analysis therefore greatly reducing measurement error. This study had a few limitations; first, this was a retrospective analysis, relied on routinely collected patient and sometimes incomplete data. Missing data on baseline and routine patient follow up characteristics likely introduced non-differential measurement bias. Some of the patients classified as LTFU may have died and this may have had an effect on the estimates, however we believe they may not have changed the general direction of the results. The age of the dataset was also a limitation. However, we believe that insights from this study are relevant. Another limitation with this analysis pertains to data quality barriers and improved data quality over time generally associated with routine program data. Routine data quality assessments and continuous quality improvement projects are ongoing to continuously improve the data and minimize missing and erroneous data. That said, these sites represent an entire region of the public sector providing ART care in Kenya.

## Conclusions

Our study demonstrated evidence of scale-up of HIV treatment programs in central Kenya. Even though majority of those enrolled were at an advanced WHO clinical stage, overall 36-month mortality remained low, though it occurred earlier during follow-up. Cohort LTFU at 36-months showed a general decline in later years and with the losses occurring within the 1st year of follow-up. Predictors of early mortality and LTFU included being male, single, separated or divorced, advanced WHO clinical stage, and among patients not on TB treatment. Evaluation of patients initiating treatment following the universal test and treat policy is recommended to evaluate any change in LTFU and mortality trends.

## Data Availability

The data used is a deidentified dataset of individual level routine HIV care and treatment data and is not currently publically available as it is property of Ministry of Health and Government of Kenya. However, the dataset can be obtained from the corresponding author based on a reasonable request.

## Abbreviations

ABC: Abacavir
ART: Antiretroviral therapy
AZT: Zidovudine
CDC: Centers for Disease Control and Prevention
d4T: Stavudine
LTFU: Loss to follow-up
NASCOP: National AIDS and STI Control Program
NCD: Noncommunicable disease
PEPFAR: President’s Emergency Plan for AIDS Relief
PITC: Provider initiated testing and counselling
PLHIV: People living with HIV
PMTCT: Prevention of mother-to-child transmission of HIV
SSA: Sub-Saharan Africa
TDF: Tenofovir
WHO: World Health Organization
